# Oral Chinese Herbal Medicine for Treatment of Dilated Cardiomyopathy: A Systematic Review and Meta-Analysis

**DOI:** 10.1155/2016/1819794

**Published:** 2016-08-18

**Authors:** Yu-Shuo Zhu, Yun-Lun Li, Jian-Qing Ju, Feng Du, Yan-Ping Zang, Xiao-Bing Wang, Jie Sheng

**Affiliations:** ^1^Traditional Chinese Medicine Clinical Research Base for Hypertension, Affiliated Hospital of Shandong University of Traditional Chinese Medicine, No. 42, Wenhua Xi Road, Jinan, Shandong 250011, China; ^2^Shandong University of Traditional Chinese Medicine, Jinan, Shandong 250355, China

## Abstract

Dilated cardiomyopathy (DCM) is one of the main causes of heart failure and could increase death, hospitalization, and rehospitalization rate. The effect of conventional medicine treatment (CMT) is limited; meanwhile, the combination of CMT and Oral Chinese Herbal Medicine (OCHM) represents exciting adjunctive therapies. In this study, we ascertained the therapeutic effect of OCHM in combination with CMT for dilated cardiomyopathy by using meta-analysis methods for controlled clinical trials. We searched studies from five databases and extracted data from these studies. We also assessed the methodological quality of the included studies. We evaluated the following outcome measures to estimate the prognosis in patients with DCM: left ventricular ejection fraction (LVEF), left ventricular end-diastolic dimension (LVEDD), stroke volume (SV), brain natriuretic peptide (BNP), 6-minute walk test (6MWT), and overall efficacy. The result showed that OCHM combined with CMT for the improvement of therapeutic effect in DCM patients. However, the evidence remains weak due to the small sample size, high clinical heterogeneity, and poor methodological quality of the included trials. Further, large sample size and well-designed trials are needed.

## 1. Introduction

Dilated cardiomyopathy (DCM), which can finally cause death by malignant ventricular arrhythmias, pump failure, or massive pulmonary embolism, is characterized by ventricular chamber enlargement and systolic dysfunction with normal LV wall thickness; usually diagnosis is made with 2-dimensional echocardiography [1]. DCM could not only obviously decrease every patients' quality of life, but also lead to higher death, hospitalization, and rehospitalization rate. The prevalence of DCM in the general population can only be estimated and clearly varies with age and geography [2]. In 1985 the National Centre for Health Statistics reported 10,345 deaths in the United States which could have been attributed to cardiomyopathy; about 87% of these were assigned to dilated cardiomyopathy [3]. In China the prevalence rate of DCM was 29.1 per 100,000 persons, the male being almost 2-fold more involved than female, and farmers accounted for 77.7% [4]. The case fatality rate of DCM was 15%~50% in 5 years [5] and brings serious burden to the society and the patients' family.

With the development of medicine, survival of adults with idiopathic DCM has improved in recent decades, with more than half surviving for 10 years [6, 7]. Conventional medicine treatment (CMT) like conventional western medicine therapy is the standardized treatment for DCM. For DCM patients' overall survival is unsatisfactory; additional therapies are necessary for this stubborn and deadly disease. Complementary and alternative medicines can perhaps benefit DCM patients as an adjunctive therapy. In China, traditional Chinese medicine (TCM) combined with conventional western medicine is diffusely used to treat DCM. From some systematic reviews and clinical researches we can discover that “Huangqi injection” [8], “Qiliqiangxin Capsule” [9], and “Shenfu injection” [10] are all effective Chinese drugs pharmaceutics for treating DCM. However, a comprehensive review of the current evidence for the effects of Oral Chinese Herbal Medicine (OCHM) on curative effect in patients with DCM is not available.

This study aims to systematically evaluate the results of RCTs investigating OCHM combined with CMT in patients with DCM using EF, LVEDD, SV, BNP, 6-minute walk test (6MWT), and overall efficacy as outcome measures.

## 2. Materials and Methods

### 2.1. Searching Strategy

Randomized controlled trials (RCTs) assessing the administration of Oral Chinese Herbal Medicine (OCHM) in the treatment for DCM were located by searching the databases CNKI, WANFANG, VIP, PubMed, and the Cochrane Controlled Trials Register and assisted by manual retrieval. There is no restriction for publication language. The databases in Chinese were searched to obtain as much as possible relevant trials, because in China OCHM is generally used, and relevant theses were intensively published in Chinese journals. The last search was run on October 2015; case reports and small case series were excluded.

PubMed searching strategy includes the following:

 #1 chinese medicine
 #2 chinese drug
 #3 herb
 #4 chinese herb
 #5 herbal medicine
 #6 chinese herbal medicine
 #7 traditional chinese medicine
 #8 TCM
 #9 Chinese herbology
 #10 OR #1–#9
 #11 dilated cardiomyopathy
 #12 DCM
 #13 OR #11-#12
 #14 #10 AND #13

The search strategy was applied to PubMed and was adapted to other forms in other databases. These terms were translated into Chinese as key words when searching Chinese databases.

### 2.2. Inclusion Criteria

Search results were screened for trials by two reviewers (Yu-Shuo Zhu and Jian-Qing Ju). Two reviewers worked independently when using the following items as essential inclusion criteria:

1. type of studies: randomized controlled trials (RCTs) with or without blinding;
2. the inclusion of cases in line with national or international standards for the diagnosis of DCM and the average age of 18 years or older;
3. trials containing patients with DCM being eligible, irrespective of the etiology, and DCM being diagnosed by the following criteria: DCM to be diagnosed according to the following accepted criteria of DCM: DCM diagnostic criteria established by WHO/ISFC [34], the concept and diagnosis criteria of DCM from textbook [35–38], and the summary of the symposium on myocarditis and cardiomyopathy in China [32, 33];
4. the New York Heart Association (NYHA) functional class at II to IV;
5. the treatment groups receiving Chinese medicine formulae together with conventional medicine treatment (CMT);
6. the control groups receiving CMT only;
7. CMT including interventions such as oxygen uptake, rest-cure, and low-salt diet, with medicines including angiotensin-converting enzyme (ACE) inhibitors, angiotensin-receptor blockers (ARBs), beta-receptor blockers, diuretics, aldosterone receptor blockers, digitalis preparation, drugs belonging to ester nitrate, and others recommended in the Chinese suggestions for diagnosis and treatment of cardiomyopathy;
8. outcome measurements including at least 2 of these parameters: ejection fraction (EF), left ventricular end-diastolic dimension (LVEDD), stroke volume (SV), BNP, 6-minute walk test, and overall efficacy as outcome measures;
9. treatment course: at least 2-week duration.

### 2.3. Exclusion Criteria

Exclusion criteria included the following items:

1. quasi-randomized trials whose methods of allocation use date of birth, date of admission, or alternation;
2. trials that included patients with acute heart failure;
3. patients whose New York Heart Association (NYHA) functional class at II to IV was caused by no myocardiac pathogeny (like hyperthyreosis, spanemia, chronic left-sided heart failure, etc.);
4. the treatment groups not receiving CMT or oral medicine;
5. the treatment groups receiving Chinese patent medicine;
6. the treatment groups receiving other traditional Chinese medicine (TCM) therapies such as acupuncture and moxibustion, massage, and ear pressure beans with or without OCHM.

### 2.4. Data Analysis

Two authors (Yu-Shuo Zhu and Jian-Qing Ju) conducted the data extraction independently. The extracted data included authors, title of study, year of publication, sample size, age and sex of the participants, diagnosis standard, risk factors for CHF, disease duration, New York Heart Association (NYHA) classification, name and component of Chinese herbs, details of the control interventions, course, follow-up, adverse effects of each study, and the details of methodological information. Disagreements were resolved by discussion, and consensus was reached through a third party (Yun-Lun Li). The methodological quality of trials was assessed independently using criteria from the Cochrane Handbook for Systematic Review of Interventions, version 5.1.0 [39]. The items included random sequence generation, allocation concealment, blinding, incomplete outcome data, selective outcome reporting, and other bias (defined as baseline data comparability). The risk of bias was categorized as low, unclear, or high.

When studies are gathered from the published literature, the random-effects model is generally a more plausible match [40]. Considering this is a more conservative method than a fixed-effect model and study heterogeneity can vary beyond chance, thus it provided a more conservative result. We used a random-effects model to estimate the overall effect when *P* < 0.1. And when *P* > 0.1, we choose the fixed-effect model. Statistical heterogeneity among trials was assessed by Cochrane's *Q* test and *I* ^2^. Meta-analysis was conducted using RevMan 5.2 (Cochrane Collaboration).

## 3. Results

### 3.1. Description of Studies

**Figure 1:**
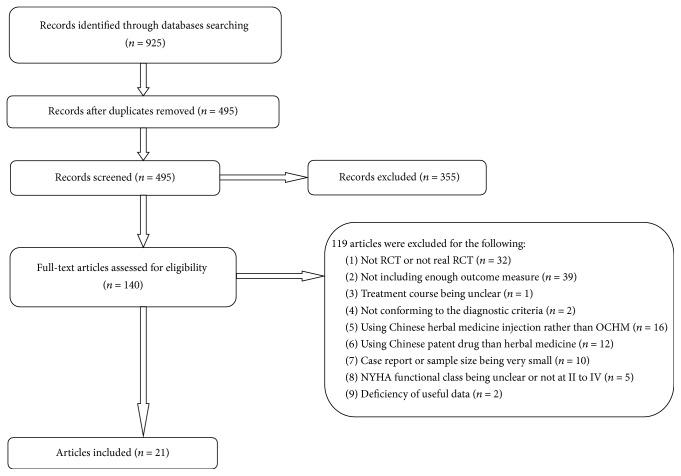
Flow chart of literature search.

After primary searches from the databases, 925 articles were screened. After removal of the duplicates, 495 records remained. After reading the titles and abstracts, 355 articles of them were excluded and full texts of 140 articles were retrieved. By reading the full texts of the remaining 140 articles, 119 articles were excluded for not meeting our inclusion criteria. Among them, 32 were excluded for not being RCT or not being real RCT, 39 for not including at least two of the parameters EF, LVEDD, SV, BNP, 6-minute walk test, and overall efficacy as outcome measures, 1 for treatment course being unclear, 2 for not conforming to the diagnostic criteria, 16 for using Chinese herbal medicine injection rather than oral administration, 12 for the treatment group receiving Chinese patent drug rather than herbal medicine; 10 for being case report or the sample size being very small, 5 for the NYHA functional class being unclear or not at II to IV, and 2 excluded due to deficiency of useful data for meta-analysis. Finally, 21 studies, involving a total of 1,566 participants, meet our inclusion criteria. The screening process was summarized in the flow diagram (Figure 1).

All the RCTs included in the meta-analysis were conducted in China and published in Chinese (the overview of the 21 studies included is indicated in Table 1). All of those studies were published in Chinese language. All studies were performed in China, and the studies involved a total of 1,566 patients with DCM. In addition, all studies exhibited comparable baseline patient characteristics, including age and gender. And there were no significant differences among them.

### 3.2. Risk of Bias Assessment

The majority of the included trials were assessed to be of poor methodological quality according to the predefined quality assessment criteria (Table 2).

**Figure 2:**
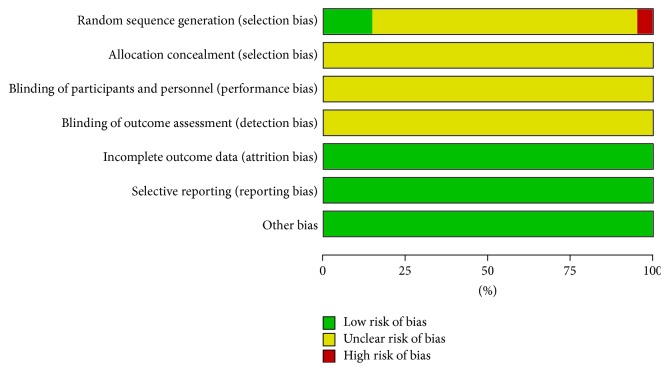
Risk of bias summary.

**Figure 3:**
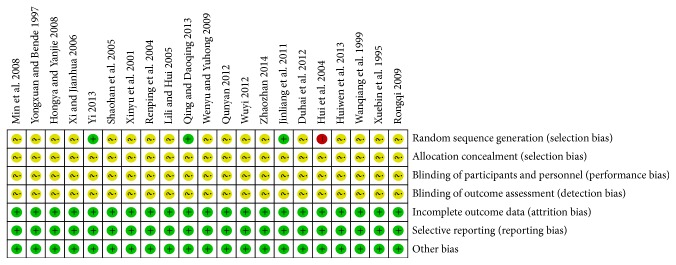
Risk of bias summary.

The randomized allocation of participants was mentioned in all trials; however, only 3 trials [17, 18, 41] stated the methods for sequence generation by random number table and one trial [23] by the order of treatment. Insufficient information was provided to judge whether or not it was conducted properly. No trials reported adequate allocation concealment. Neither double-blinding nor single-blinding was applied in these trials. None of the trials had a pretrial estimation of sample size. Five trials [21, 23, 26, 30, 31] mention follow-up. No trials reported dropout or withdrawal; meanwhile, none of them reported whether they had used intention-to-treat (ITT) analysis. All trials provided baseline data for the comparability among groups. The results of the assessment of risk of bias were presented in “risk of bias summary” figures produced by RevMan 5.2 (Figures 2 and 3).

### 3.3. Clinical Effect

#### 3.3.1. Decrease of BNP

**Figure 4:**
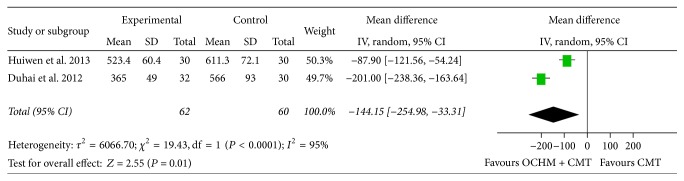
Forest plot of decrease of patients' BNP.

Figure 4 presents the findings of the 2 trials included for meta-analysis. For the 2 trials, OCHM plus CMT was found to be a significant method of decreasing plasma brain natriuretic peptide (BNP) when compared to the control group. Meta-analysis shows a significant improvement in BNP [MD = −144.15, 95% CI [−254.98, −33.31], and *P* = 0.01], which means that OCHM plus CMT is significantly better than CMT in decreasing BNP of heart. Highly significant heterogeneity was found among these 2 studies (*χ* ^2^ = 19.43, *I* ^2^ = 95%, and *P* < 0.0001), indicating their heterogeneity.

#### 3.3.2. Improvement of LVEDD, EF, and SV

**Figure 5:**
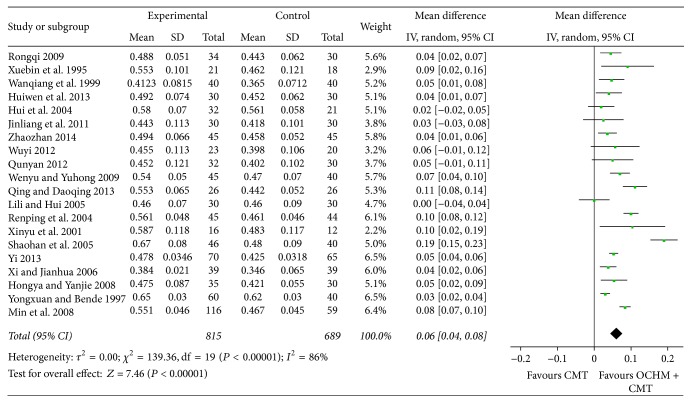
Forest plot of improvement of patients' LVEF.

The random-effects model was used for statistical analysis as the heterogeneity was expected. Among the 20 studies involving left ventricular ejection fraction (LVEF), OCHM plus CMT was found to be a significant method to improve left ventricular function when compared to CMT. Meta-analysis shows a significant improvement in EF [MD = 0.06, 95% CI [0.04, 0.08], and *P* < 0.0001], which means that OCHM plus CMT is significantly better than CMT in improving ventricular ejection fraction. Highly significant heterogeneity was found among these 20 studies (*χ* ^2^ = 139.36, *I* ^2^ = 86%, and *P* < 0.00001), indicating their heterogeneity (Figure 5).

**Figure 6:**
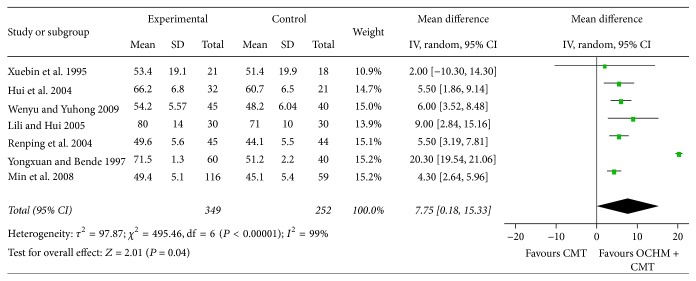
Forest plot of improvement of patients' SV.

For the 7 studies mentioning stroke volume (SV), meta-analysis shows a significant improvement in SV [MD = 7.75, 95% CI [0.18, 15.33], and *P* = 0.04], which means that OCHM plus CMT is significantly better than CMT in increasing SV. Highly significant heterogeneity was found among these 7 studies (*χ* ^2^ = 495.46, *I* ^2^ = 99%, and *P* < 0.00001), indicating their heterogeneity (Figure 6).

**Figure 7:**
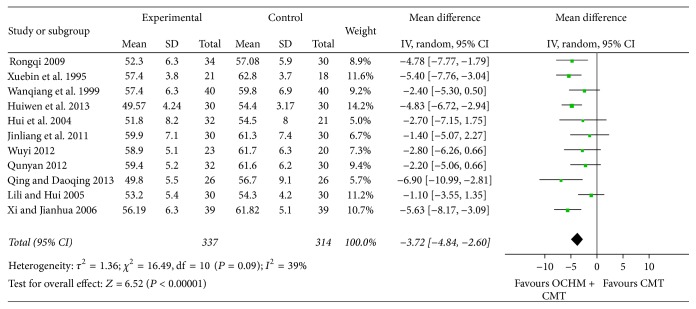
Forest plot of improvement of patients' LVEDD.

Meta-analysis shows a significant improvement in LVEDD [MD = −3.72, 95% CI [−4.84, −2.60], and *P* < 0.00001], which means that OCHM plus CMT is significantly better than CMT in narrowing left ventricular end-diastolic dimension. Low heterogeneity was found among these 11 studies (*χ* ^2^ = 16.49, *I* ^2^ = 39%, and *P* = 0.09) (Figure 7).

#### 3.3.3. 6MWT

**Figure 8:**
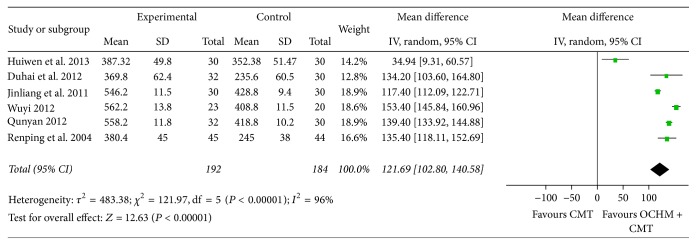
Forest plot of improvement of patients' 6MWT.

15 studies comparing OCHM plus CMT versus CMT found that OCHM plus CMT achieved a greater improvement than using CMT only [MD = 121.69, 95% CI [102.80, 140.58], and *P* < 0.00001], which means that OCHM plus CMT is significantly better than CMT in increasing the exercise tolerance. Highly significant heterogeneity was found among these 6 studies (*χ* ^2^ = 121.97, *I* ^2^ = 96%, and *P* < 0.00001). The results of the meta-analysis are shown in Figure 8.

#### 3.3.4. Overall Efficacy

**Figure 9:**
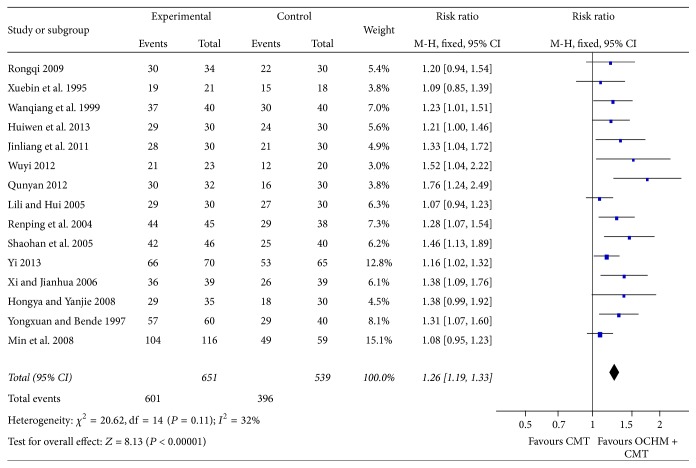
Forest plot of overall efficacy.

In this meta-analysis we chose the fixed-effect model, and it shows a significant improvement in overall efficacy [RR = 1.26, 95% CI [1.19, 1.33], and *P* < 0.00001], which means that OCHM plus CMT is significantly better than CMT in acquiring better curative effect (Figure 9).

## 4. Discussion

DCM is a kind of refractory cardiomyopathy and its cause is still not clear; the majority of Chinese and foreign scholars believe that the occurrence and development of DCM rely on the resulting myocardial autoimmune injury and the persistent viral infection [42]. Based on CMT, although the long-term survival rate of patients has been improved, because of the complexity of the pathological and physiological mechanism of DCM itself, the treatment method has not yet achieved satisfactory clinical results. As a complementary and alternative therapy, TCM has increasingly drawn a wider range of interest among DCM studies because it can increase the efficacy when combined with CMT.

Echocardiograph is an effective adjunct for cardiac function or structure assessment in cardiomyopathy diagnosis and evaluation. Many experiments show that the degree of left ventricular dysfunction is the most important prognostic factor of DCM patients. Parameters like left ventricular ejection fraction (LVEF), left ventricular end-diastolic dimension (LVEDD), and stroke volume (SV) reflect left ventricular function and size. Left ventricular ejection fraction (LVEF) provides useful diagnostic and prognostic information for patients with idiopathic dilated cardiomyopathy [5, 41]. Stroke volume (SV) and its derivative index are the result of left ventricular preload, afterloading, and myocardial contractility's synthetic action. This type of index is significantly affected by left ventricular preload and afterloading. 6MWT, a submaximal exercise test, simply measures the distance ambulated on a level hallway surface during 6 min [43]. The 6MWT is a simple clinical tool to assess cardiac functional capacity and to predict patient's status. The Seattle Heart Failure Study has shown that serum parameters can be as important as echocardiography in predicting outcomes and also add BNP to the model to perfect the prognostic power of the score [44].

### 4.1. Main Findings

21 RCTs and 1,566 participants were included in this review. Our meta-analysis of the overall effect rate found that OCHM combined with CMT were more effective than CMT for the treatment of DCM. This study also shows that the use of oral herbal medicine for the treatment of DCM based on CMT can provide a satisfactory therapeutic effect to patients with different gender, different age, and different degree, by improving the cardiac function and regulating atrioventricular size, and improve the exercise tolerance of patients. From this we can infer that OCHM plays a similar role in enhancing myocardial contractility, diuretic, dilation of blood vessels, antimyocardial ischemia, inhibiting myocardial remodeling, and so on, which can be similar to CMT.

### 4.2. About OCHM

The efficacy of different drugs used in these experiments is mainly reflected in improving myocardial contractile function, improving myocardial metabolism, improving vascular endothelial function, antiplatelet aggregation, regulating the immune function, improving the body's resistance, protecting ischemic and hypoxia myocardium, clearing virus persistent infection, and so on.

Modern pharmacological studies confirmed that Danshen can increase plasma CGRP, reduce plasma endothelin levels, reduce the plasma and myocardial local Ang II, and promote myocardial repair [45]. Huangqi could promote cell metabolism, improve the ability of myocardial hypoxia tolerance [46], reduce rennin-angiotensin and brain natriuretic peptide level [47], reduce the preload and afterload, enhance myocardial contractility, and improve heart function from many aspects. Gegen has the function of expanding peripheral blood vessels and contains puerarin which can further reduce the level of serum BNP in patients with heart failure [48]. The effective components of Chuanxiong (like ligustrazine and ferulic acid) have the function of vasodilation, improving microcirculation, and antimyocardial ischemia. Renshen, Tinglizi can enhance myocardial contractility and increased cardiac output function [49, 50]. In addition, astragaloside can promote macrophages' synthesis of primary lysosomes and regulate secretion of IL-1 in macrophages [42, 51]. Ginsenoside can reduce the open probability of B type, L type, and T type calcium ion in rats' myocardial cells, shorten the opening time, promote the gene expression of lymphocyte IL-1 and IL-6 [42, 51]. Huangqi has antiviral and immune regulation effects and improves heart function [52].

### 4.3. Limitations of This Review

The included studies had limitations in methodological qualities. Only 3 of the trials reported on how the participants are randomly assigned to the intervention groups. The other trials simply mention “randomization,” with none of the trials mentioning the use of allocation concealment and blinding. Five of the trials mention follow-up.

Overview of the literature collected by this study: although results showed that OCHM in patients with DCM have significantly increased left ventricular ejection function, delay ventricular enlargement, and improve exercise tolerance, the literature quality is still low.

This study suggests that oral herbal medicine can effectively improve the cardiac function of patients with DCM, but the results have very significant heterogeneity; the reason may be that the quality of current domestic clinical research is not high. What is more, the clinical samples are small, the observation time is short, and the relevant evidence-based data needs further observation; all of these items restrict the promotion of the level.

This affects the evaluation objective effect in a certain extent. We expect to carry out higher standard randomized clinical trial and mechanism revealing of oral herbal medicine, in order to overcome the DCM, a severe case in the modern medicine.

## 5. Conclusions

In recent years, system evaluation and meta-analysis have been widely used in clinical medicine, public health, molecular genetics, drug evaluation, medical insurance, medical education, and many other biomedical fields. Meta-analysis can be used to increase the sample size and improve the efficacy of the test, especially when multiple results are inconsistent or not statistically significant. Meta-analysis can be used to get more close to the real situation of the comprehensive results [53].

The combination of oral herbal medicine and CMT can be used as an effective therapy of DCM; the specific mechanism needs further study.

From the traditional Chinese medicine diagnosis and treatment based on syndrome differentiation to confirm its own theoretical advantages, we can clearly conclude that the effective law and scientific induction of the high repeatability of the treatment experience still need to be further explored.

## Figures and Tables

**Table 1:** An overview of the included studies.

References	Number of participants	Number of males/females	Diagnosis standard	Age (years)	Disease duration	NYHA classification	Intervention	Control	Course	Outcome measures
Duhai et al. 2012 [11]	T 32/C 30	T 22/10 C 18/12	Internal medicine	T 47.3 ± 4.2 C 45.9 ± 4.6	T 12 ± 6.5 m C 13 ± 7.2 m	III-IV	Baihuasheshecao, Shichangpu, Danshen, Dilong, Taizishen, Chishao, Huangqi, Gegen, Danggui, Danpi, Sharen, Daqingye, Quanxie, Caodoukou + CMT	CMT + Qishenyiqi dripping pills	2 M	BNP, 6MWT

Zhaozhan 2014 [12]	T 45/C 45	T 35/10 C 36/9	Internal medicine	T 57.3 ± 6.2 C 57.8 ± 5.8	T 3.0 ± 0.3 y C 3.0 ± 0.2 y	II–IV	Baihuasheshecao, Shichangpu, Danshen, Dilong, Taizishen, Chishao, Gegen, Huangqi, Quanxie, Danggui, Danpi, Sharen, Daqingye, Caodoukou + CMT	CMT	2 M	BNP, LVEF

Wenyu and Yuhong 2009 [13]	T 45/C 40	T 29/16 C 25/15	WHO/ISFC	T 16–70, M = 41 C 17–71, M = 40	Unclear	II–IV	Fuzi, Baizhu, Fuling, Baishao, Danggui, Taoren, Guizhi, Huangqi, Renshen, Zhigancao, Honghua, Shengjiang, Rougui + CMT	CMT	6 W	LVEF, SV

Qing and Daoqing 2013 [14]	T 26/C 26	T 12/14 C 11/15	Chinese meeting summary^*∗*^	T 46 ± 5.6 C 44 ± 4.8	T 8.8 ± 5.6 y C 7.6 ± 5.2 y	II–IV	Huangqi, Dangshen, Danshen, Yiyiren, Fuzi, Zhigancao, Guizhi, Chuanxiong, Fuling, Zelan, Zexie, Tinglizi, Jiangxiang + CMT	CMT	2 M	LVEF, LVEDD

Yi 2013 [15]	T 70/C 65	T + C 92/43	Practical Department of Internal Medicine	T 45 ± 7.5 C 44.5 ± 9	Unclear	III-IV	Fuling, Zhuling, Zexie, Tinglizi, Baishao, Baizhu, Fuzi, Shuizhi, Huangqi, Dangshen, Guizhi + CMT	CMT	2 W	LVEF, overall efficacy

Min et al. 2008 [16]	T 116/C 59	T 61/55 C 33/26	WHO/ISFC	T 6–80, M = 43.1 C 8–75, M = 39	T 3 M–10 Y, M = 4.9 Y C 6 M–9 Y, M = 4.6 Y	II–IV	Huangqi, Sanqi, Danshen, Shengdihuang, Huanglian, Lianqiao, Guizhi, Fuling, Danpi + CMT	CMT	300 D	LVEF, SV, overall efficacy

Xinyu et al. 2001 [17]	T 16/C 12	T 9/7 C 7/5	Chinese meeting summary	T 22–60 Y C 28–62 Y	Unclear	II–IV	Huangqi, Fuzi, Guizhi, Fangji, Dangshen, Fuling, Danshen, Yuzhu, Chishao, Buguzhi, Baizhu + CMT	CMT	2 M	LVEF

Hongya and Yanjie 2008 [18]	T 35/C 30	T 21/14 C 19/11	WHO/ISFC	T 30–61 Y, M = 41 C 28–63 Y, M = 42	Unclear	II–IV	Renshen, Guizhi, Maidong, Shengdihuang, Ejiao, Huomaren, Muxiang, Yuanzhi, Wuweizi + CMT	CMT	12 M	LVEF, overall efficacy

Lili and Hui 2005 [19]	T 30/C 30	T 18/12 C 16/14	Chinese meeting summary	T 16–60 Y, M = 38.2 C 18–58 Y, M = 37.5	T 1.5–10 Y, M = 4.5 C 1–11 Y, M = 4.2	II-III	Huangqi, Taoren, Renshen, Tinglizi, Fuling, Danshen, Wujiapi, Guizhi, Fuzi, Honghua +CMT	CMT	1 M	LVEF, SV, LVEDD, overall efficacy LVEDD

Hui et al. 2004 [20]	T 32/C 21	T 20/12 C 13/8	WHO/ISFC	T 41.2 ± 12.2 C 42.3 ± 15	T 38.6 ± 14.1 M C 40.86 ± 16 M	II–IV	Huangqi, Renshen, Maidong, Wuweizi, Guizhi, Baizhu, Fuling, Wujiapi, Zelan, Danshen, Gegen, Yuzhu, Xianlingpi, Zhigancao + CMT	CMT	3 M	LVEF, SV

Rongqi 2009 [21]	T 34/C 30	T 24/10 C 22/8	Chinese meeting summary	T 30–63 Y, M = 40.2 C 35–62 Y, M = 39.5	T 6 M–7 Y C 5 M–8 Y	II–IV	Renshen, Shuizhi, Zhigancao, Guizhi, Maidong, Shengdihuang, Gansong, Chuanxiong, Tinglizi, Zexie + CMT	CMT	6 M	LVEF, LVEDD, overall efficacy

Xi and Jianhua 2006 [22]	T 39/C 39	T 29/10 C 28/11	WHO/ISFC	T M = 43.6 C M = 46.8	T M = 632 D C M = 647 D	II–IV	Huangqi, Renshen, Danshen, Fuzi, Maidong, Tanxiang, Wuweizi, Zelan Danggui + CMT	CMT	3 M	LVEF, LVEDD, overall efficacy

Renping et al. 2004 [23]	T 45/C 44	T 29/16 C 26/18	WHO/ISFC	T 7–72, M = 40.2 C 10–69, M = 38.1	T 0.5–10, M = 4.8 Y C 0.8–9, M = 4.5 Y	III-IV	Huangqi, Dangshen, Guizhi, Chuanxiong, Danshen, Sanqi, Huanglian, Lianqiao + CMT	CMT	1 Y	6MWT, LVEF, SV, overall efficacy

Shaohan et al. 2005 [24]	T 46/C 40	T 29/17 C 24/16	WHO/ISFC	T 2–70, M = 39 C 2–69, M = 41	Unclear	II–IV	Huangqi, Dangshen, Baizhu, Zhigancao, Danggui, Shengma, Chaihu, Fuling, Danshen, Suanzaoren, Juhong, Zhiqiao, Zhuru, Banxia + CMT	CMT	8 W	LVEF, overall efficacy

Yongxuan and Bende 1997 [25]	T 60/C 40	T 36/24 C 26/14	Practical Department of Internal Medicine	Unclear	Unclear	II–IV	Taizishen, Maidong, Wuweizi, Fuling, Zhuling, Baizhu, Guizhi, Zexie, Suanzaoren, Yuanzhi, Longgu, Muli, Zhigancao + CMT	CMT	2 W	LVEF, SV, overall efficacy

Xuebin et al. 1995 [26]	T 21/C 18	T 19/2 C 15/3	WHO/ISFC	T 16–67, M = 42 C 24–60, M = 47	T 1–9 Y, M = 4.3 C 10 M–7 Y, M = 3.9	II–IV	Huangqi, Danshen, Fuzi, Chuanxiong, Xianlingpi + CMT	CMT	2 W	LVEF, SV, LVEDD, overall efficacy

Wanqiang et al. 1999 [27]	T 40/C 40	T 26/14 C 24/16	WHO/ISFC	T 18–62, M = 39.8 C 17–66, M = 40.3	Unclear	II–IV	Renshen, Huangqi, Danggui, Chuanxiong, Baizhu, Maidong, Wuweizi, Suanzaoren, Boziren, Yuanzhi, Zhiqiao, Fuling, Fushen, Banxia, Zhigancao + CMT	CMT	4 W	LVEF, LVEDD, overall efficacy

Huiwen et al. 2013 [28]	T 30/C 30	T + C 42/18	Internal Medicine	45 ± 4.8	14 ± 8.5 M	II–IV	Changpu, Danshen, Baihuasheshecao, Taizishen, Chishao, Huangqi, Gegen, Danggui, Danpi, Daqingye, Quanxie, Dilong, Sharen, Caodoukou + CMT	CMT	2 M	BNP, 6MWT, LVEF, LVEDD, overall efficacy

Wuyi 2012 [29]	T 23/C 20	T 13/10 C 11/9	Chinese meeting summary	T 46–77, M = 48.6 C 40–78, M = 46.7	T 1.5–10 Y, M = 3.2 C 1-9 Y, M = 3.8	II–IV	Huangqi, Fuling, Danggui, Renshen, Maidong, Wuweizi, Fuzi, Rougui, Guizhi, Baizhu, Zexie, Tinglizi, Danshen + CMT	CMT	90 D	6MWT,LVEF, LVEDD, overall efficacy

Qunyan 2012 [30]	T 32/C 30	T 20/12 C 19/11	WHO/ISFC	T 41–78, M = 47.8 C 40–73, M = 45.6	T 1–11 Y, M = 3.1 C 1–12, M = 3.2	II–IV	Gancao, Renshen, Shengjiang, Guizhi, Shengdihuang, Shuizhi, Tinglizi, Ejiao, Maidong, Huomaren, Dazao + CMT	CMT	3 M	6MWT, LVEF, LVEDD, overall efficacy

Jinliang et al. 2011 [31]	T 30/C 30	T 27/3 C 26/4	Chinese meeting summary	T 40 ± 5 C 41 ± 6	Unclear	II–IV	Huangqi, Danggui, Renshen, Maidong, Wuweizi, Guizhi, Fuling, Zexie, Yimucao, Tinnglizi, Baizhu, Chenpi, Dazao, Shenggancao + CMT	CMT	90 D	6MWT, LVEF, LVEDD, overall efficacy

^*∗*^Chinese meeting summary: summary of the symposium on myocarditis and cardiomyopathy in China [32, 33].

**Table 2:** The methodological quality of included trials.

References	Random sequence generation	Allocation concealment	Blinding	Incomplete outcome data	Selective outcome reporting	Baseline data comparability	Follow-up
Duhai et al. 2012 [11]	Unclear	Unclear	Unclear	No	No	Yes	No
Zhaozhan 2014 [12]	Unclear	Unclear	Unclear	No	No	Yes	No
Wenyu and Yuhong 2009 [13]	Unclear	Unclear	Unclear	No	No	Yes	No
Qing and Daoqing 2013 [14]	Random number table	Unclear	Unclear	No	No	Yes	No
Yi 2013 [15]	Random number table	Unclear	Unclear	No	No	Yes	No
Min et al. 2008 [16]	Unclear	Unclear	Unclear	No	No	Yes	No
Xinyu et al. 2001 [17]	Unclear	Unclear	Unclear	No	No	Yes	No
Hongya and Yanjie 2008 [18]	Unclear	Unclear	Unclear	No	No	Yes	Yes
Lili and Hui 2005 [19]	Unclear	Unclear	Unclear	No	No	Yes	No
Hui et al. 2004 [20]	Order of treatment	Unclear	Unclear	No	No	Yes	Yes
Rongqi 2009 [21]	Unclear	Unclear	Unclear	No	No	Yes	No
Xi and Jianhua 2006 [22]	Unclear	Unclear	Unclear	No	No	Yes	No
Renping et al. 2004 [23]	Unclear	Unclear	Unclear	No	No	Yes	Yes
Shaohan et al. 2005 [24]	Unclear	Unclear	Unclear	No	No	Yes	No
Yongxuan and Bende 1997 [25]	Unclear	Unclear	Unclear	No	No	Yes	No
Xuebin et al. 1995 [26]	Unclear	Unclear	Unclear	No	No	Yes	No
Wanqiang et al. 1999 [27]	Unclear	Unclear	Unclear	No	No	Yes	Yes
Huiwen et al. 2013 [28]	Unclear	Unclear	Unclear	No	No	Yes	Yes
Wuyi 2012 [29]	Unclear	Unclear	Unclear	No	No	Yes	No
Qunyan 2012 [30]	Unclear	Unclear	Unclear	No	No	Yes	No
Jinliang et al. 2011 [31]	Random number table	Unclear	Unclear	No	No	Yes	No
